# The Production of Biochar and Its Impact on the Removal of Various Emerging Pollutants from Wastewater: A Review

**DOI:** 10.3390/toxics13121079

**Published:** 2025-12-15

**Authors:** Zafran Ullah, Collin G. Joseph, Zhen-Yu Tian, Muhammad Yasin, Muhammad Naeem Khan, Sajid Ali, Aqsa Khan, Jonathan Suazo-Hernández, Patricia Poblete-Grant, Muhammad Ikram Nabeel

**Affiliations:** 1 Department of Engineering, School of Engineering and Technology, Sunway University, Bandar Sunway 47500, Selangor, Malaysia; zafranktk69@gmail.com; 2 Sonophotochemistry Research Group, Faculty of Science and Technology, Universiti Malaysia Sabah, Kota Kinabalu 88400, Sabah, Malaysia; ch.ikramnabeel802@gmail.com; 3 Industrial Chemistry Programme, Faculty of Science and Technology, Universiti Malaysia Sabah, Kota Kinabalu 88400, Sabah, Malaysia; 4 Institute of Engineering Thermophysics, Chinese Academy of Sciences, Beijing 100190, China; tianzhenyu@iet.cn; 5 Institute of Innovation Materials and Energy, School of Chemistry and Chemical Engineering, Yangzhou University, Yangzhou 225002, China; yasinkhan1503036@gmail.com; 6 Department of Chemistry, Allama Iqbal Open University, Islamabad 44000, Pakistan; 7 CAS Key Laboratory of Urban Pollutant Conversion, Institute of Urban Environment, Chinese Academy of Sciences, Xiamen 361021, China; sajid@iue.ac.cn (S.A.); aqsa@iue.ac.cn (A.K.); 8 Facultad de Medicina Veterinaria y Agronomía, Universidad de Las Américas, Sede Concepción, Concepción 4030000, Chile; 9 Department of Chemical Sciences and Natural Resources, Universidad de La Frontera, Avenida Francisco Salazar 01145, Temuco P.O. Box 54-D, Chile; 10 Facultad de Medicina Veterinaria y Agronomía, Universidad de Las Américas, Campus Providencia, Manuel Montt 948, Santiago 75009, Chile; ppobleteg@udla.cl

**Keywords:** biochar, sustainable agriculture, environmental remediation, adsorption, functional modification, circular economy

## Abstract

Recent advances in agricultural biotechnology and sustainable farming have drawn attention to biochar as a multifunctional material for environmental remediation. Among its emerging applications, biochar has demonstrated remarkable potential in wastewater treatment, particularly as an efficient and sustainable adsorbent for pollutant removal. Numerous studies over the past decades have highlighted its effectiveness in eliminating a wide range of contaminants. This efficiency is mainly due to its abundant feedstock availability, simple production processes, and favorable surface and structural properties. This review summarizes current developments in biochar use for wastewater treatment, emphasizing its adsorption capabilities and the underlying mechanisms responsible for pollutant removal. Key modification strategies, physical, chemical, and biological, are discussed in detail to illustrate how biochar performance can be optimized for specific treatment goals. Furthermore, the prospects of biochar-based technologies are explored, with a focus on their role in addressing both inorganic and organic pollutants. This review also describes the use of biochar in adsorbing metals, organic contaminants, and industrial waste. The integration of biochar into sustainable water management systems presents a promising pathway toward achieving long-term environmental and agricultural resilience.

## 1. Introduction

**Figure 1:**
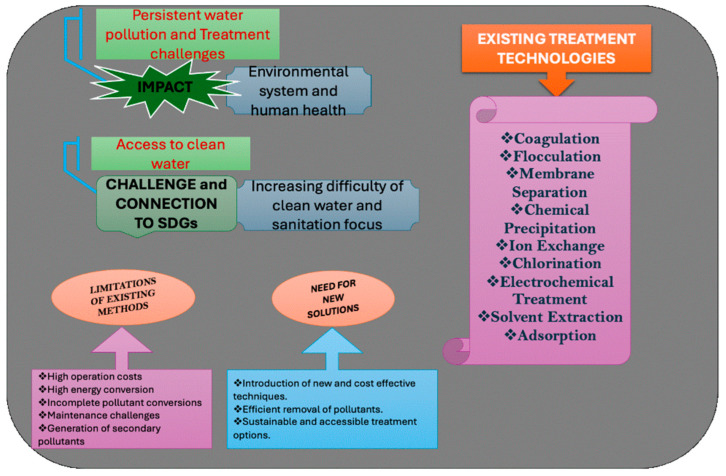
Existing technologies and the challenges in treating water pollution.

The rapid growth of population, industrialization activities, along with changes in how people consume goods, has led to an increase in the number of emerging pollutants (EPs) in both water and soil environments [1,2]. The problem of water pollution is now considered an alarming situation for various environmental matrices including human beings [3,4]. The availability and access to clean and safe drinking water have now become a challenging task, although the Sustainable Development Goals (SDGs) have proposed an agenda about clean water and sanitation [5,6,7]. The emergence of various threatening pollutants such as heavy metals, pesticides, organics, dyes, pharmaceutical waste, antibiotics, and surfactants from industrial, agricultural, and household activities represents one of the most significant groups of pollutants discharged into aquatic systems [6,8]. Even at low concentrations, these pollutants can have a strong effect on marine life [4,9]. In past research, many technologies like coagulation, flocculation, membrane separation, chemical precipitation, ion exchange, chlorination, demulsification electrochemical treatment, solvent extraction, and adsorption have been adopted for wastewater treatment, making them fit for drinking and irrigation purposes [10,11,12]. Different catalysts are also employed for this purpose and various catalysts (SiO_2_, CeO_2_, CdS, Fe_2_O_3_, SnO_2_, Fe_3_O_2_, NiO, TiO_2_, GO, g-C_3_N_4_, ZnO, ZnS, BiOI, 2D MXenes, and WO_3_) are used for the removal of pollutants [6,8,12,13,14,15,16]. All of these have been widely applied for the degradation or adsorption of multiple contaminants. These various catalysts offer highly active surfaces and adaptable physicochemical properties. These characteristics allow them to interact strongly with pollutants and can effectively remove them through various processes (adsorption, photocatalytic reactions, and oxidation-reduction mechanisms). Nevertheless, these existing methods are costly, do not sufficiently convert pollutants to fewer toxic forms, and have high operating costs, maintenance, high energy consumption, and a tedious reaction process (Figure 1 represents the existing technologies for treating water pollution and its challenges). Also, they are unable to remediate the secondary pollutants arising from primary reactions in water bodies [11,17]. The growing awareness of environmental issues has encouraged the scientific community around the world to find practical ways to reduce environmental pollution challenges [18]. Therefore, the introduction of new, efficient, and easily available low-operation techniques is highly required for the abatement of pollutants from wastewater [17,19]. Now many researchers are moving towards developing low-cost, affordable, sustainable, and effective adsorbent materials that can help clean up the environment. Adsorption technologies have gained popularity due to their simplicity and efficiency, particularly with the use of biochar as an adsorbent. Biochar, produced through the pyrolysis of biomass residues, is both environmentally friendly and cost-effective [17,20]. Its high carbon content, nutrient richness, specific surface area, and cation-exchange capacity make it an excellent candidate for removing pollutants from wastewater [21]. The effectiveness of biochar as an adsorbent is influenced by various physicochemical characteristics, including its porous structure, elemental composition, and surface functional groups. These properties are largely determined by the feedstock used and the conditions during pyrolysis [22]. Before application, it is crucial to assess the biochar’s specific surface area, pore volume, pH, and cation-exchange capacity. Despite its advantages, biochar performance can be limited by interactions with secondary molecules and challenges in separation from the treated medium. To enhance its stability and reactivity, further modifications are necessary, which can improve biochar regeneration capacity and overall effectiveness in adsorption processes.

The main aim of this review is to provide a clear understanding of how biochar is produced, its characteristics, surface functional group properties, modification of biochar, and its role in improving adsorption performance. The review also highlights the mechanisms involved in pollutant removal and how its characteristics influence the removal of various emerging pollutants from wastewater. In addition, the challenges and future opportunities for developing efficient biochar-based treatment systems are discussed. By using natural resources in environmentally friendly ways, this research contributes to the advance of green technologies, the recovery of useful materials that help minimize pollution, and the improvement of available materials. It also supports several SDGs, such as SDG-6 (Clean Water and Sanitation), SDG-9 (Industry, Innovation, and Infrastructure), and SDG-12 (Responsible Consumption and Production) [23,24]. Finally, this review discusses the future directions and practical applications of biochar in removing pollutants and purifying water. The future directions also highlight its potential as a sustainable and efficient material for cleaning up different types of environmental pollution.

## 2. Characteristics of Biochar for Adsorption

**Figure 2:**
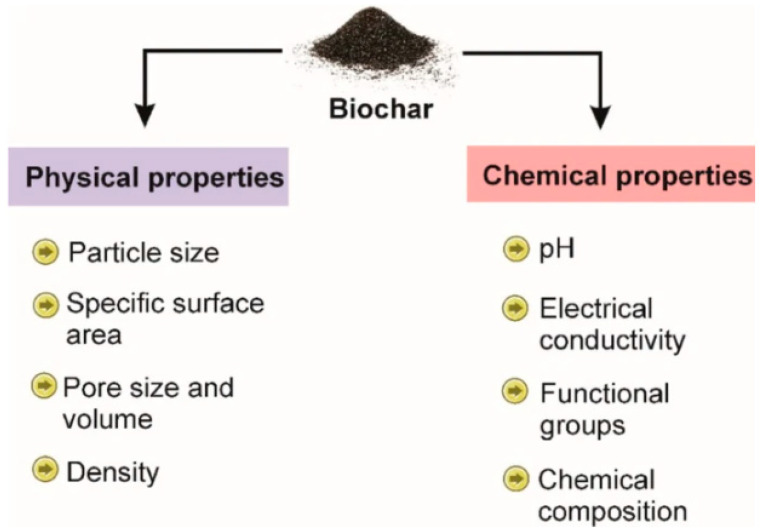
Properties of engineered biochar (reproduced with permission, Copyright 2024, *Springer Nature*) [25].

Research has extensively examined the relationships between biochar characteristics and adsorption efficiency, revealing that several features significantly influence its performance. The properties of biochar are outlined in Figure 2. The following characteristics should be kept in mind while working on biochar (surface area, porosity, pore size distribution, functional groups, cation-exchange capacity (CEC), elemental composition, pH, and hydrophobicity/hydrophilicity of biochar). A higher specific surface area provides more active sites for adsorbing pollutants, enhancing overall adsorption capacity. The porous structure of biochar facilitates the movement of pollutants into its interior, allowing for greater interaction and adsorption. The size and distribution of pores determine the types of molecules that can be effectively adsorbed, impacting overall efficiency. The assessment of porosity is crucial for determining the adsorption capacities of biochar, particularly in pollutant remediation. Research has shown a linear relationship between the adsorption capacity of toluene and the specific surface area of biochar materials; as the specific surface area increases, more adsorption sites become available, enhancing the biochar’s ability to capture and remediate pollutants.

**Figure 3:**
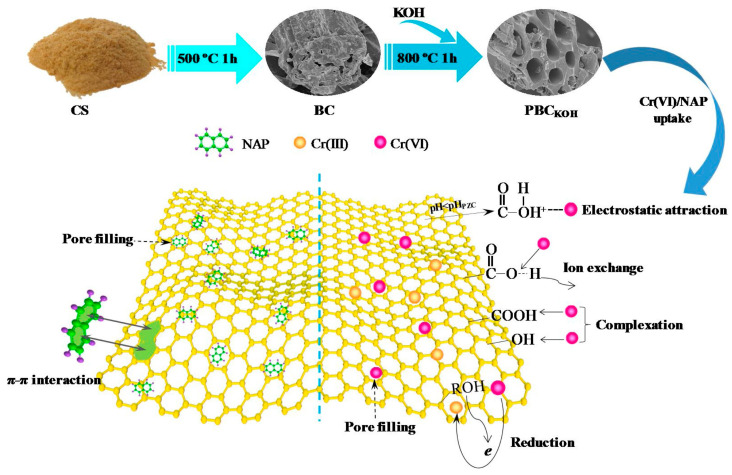
Schematic illustration of synthesis of high-performance porous biochar and the adsorption mechanisms of Cr(VI) and NAP on PBC(KOH). Reproduced with permission, Copyright 2021, *Elsevier* [29].

Various methods have been developed to increase pore volume during the pyrolysis of feedstocks, which is key for optimizing biochar characteristics [26]. Among these, physical activation methods, such as raising the pyrolysis temperature, facilitate the decomposition of biomass and the release of volatiles (such as CO, CO_2_, CH_4_, and H_2_O), thereby creating a more porous structure. For instance, temperatures exceeding 500 °C can significantly increase the porosity and surface area of the resulting biochar. In contrast, chemical activation methods also effectively enhance porosity. The addition of chemical activators (like aqueous solutions of sulfuric acid (H_2_SO_4_), phosphoric acid (H_3_PO_4_), potassium hydroxide (KOH), and sodium hydroxide (NaOH)) can lead to varying degrees of pore volume enhancement. These chemicals not only contribute to the creation of pores but also can influence the surface chemistry of biochar, potentially improving its adsorption characteristics. Notably, studies have indicated that Fe-modified biochar exhibits a higher degree of nitrobenzene mineralization compared to both Zn-modified and pristine biochar. This improvement is attributed to the enhanced porosity and surface functional groups provided by the iron modification, which facilitates better interaction with pollutants [27,28]. Such findings underscore the importance of both physical and chemical modifications in tailoring biochar for effective environmental remediation applications. Jianhua Qu synthesized the high-performance PBC (porous biochar) by a two-step pyrolysis process using corn straw. This was chemically activated by KOH, and the resulting material was utilized for the elimination of both hexavalent chromium Cr(VI) and naphthalene (NAP) from aqueous solutions. Due to the effective KOH activation, the PBC(KOH) exhibited an exceptionally large specific surface area of 2183.80 m^2^/g as well as microspores (average particle size of 2.75 nm) and main pore diameters ranging from 1 to 2 nm. The adsorbent PBC (KOH) demonstrated outstanding removal efficiency, with a theoretical monolayer uptake of 116.97 mg/g for Cr(VI). In addition, it exhibited a heterogeneous adsorption capacity of 450.43 mg/g for NAP. Equilibrium was reached within 120 min for Cr(VI) and 180 min for NAP. Further investigation into the adsorption mechanisms revealed that the Cr(VI) binding onto PBC(KOH) was mainly due to ion exchange, electrostatic attraction, reduction, and surface complexation. In contrast, the adsorption of NAP was dominated primarily by pore filling effects and π–π stacking interactions [29]. Figure 3 illustrates the proposed adsorption mechanisms of Cr(VI) and NAP by PBC(KOH). To optimize biochar adsorption capacity, it is essential to understand and enhance these characteristics through a careful selection of feedstock and pyrolysis conditions. The investigation of the relationships between biochar characteristics and the adsorption process has been widely studied. Many characteristics of the biochar have a linear response with adsorption efficiency. Therefore, it is necessary to employ the role of the chief features for boosting biochar adsorption capacity.

**Figure 4:**
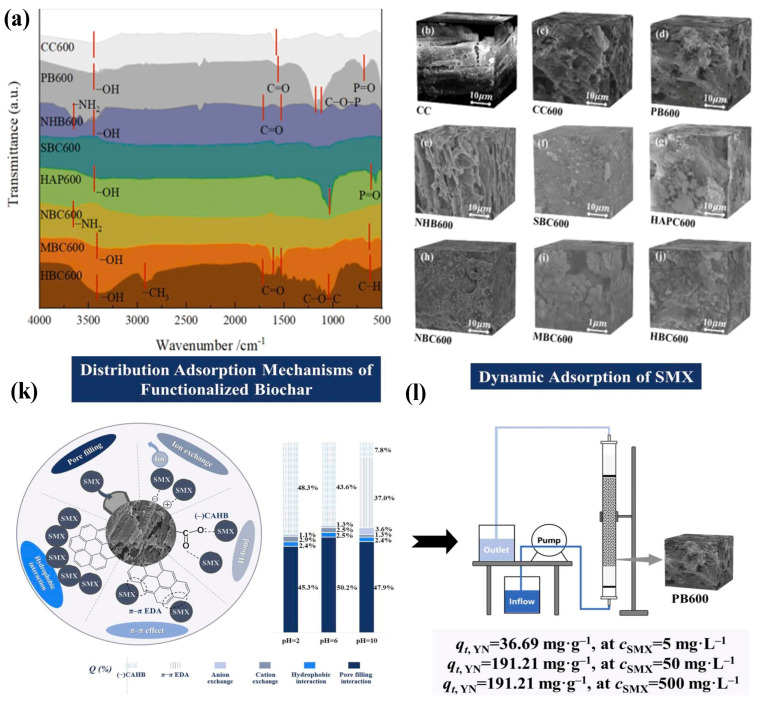
(**a**) FTIR spectra and (**b**–**j**) SEM of various functionalized biochars; (**k**) assignment of adsorption mechanisms for functionalized biochars; (**l**) dynamic absorption of SMX (reproduced with permission, Copyright 2025, *Elsevier*) [33].

Similarly, surface functional groups (hydroxyl, carboxyl) can enhance the adsorption of various contaminants through ionic and hydrogen bonding [30]. The presence of surface functional groups on biochar significantly influences its ability to adsorb contaminants at various adsorption sites. However, the precise identification of these functional groups remains a challenge. The types and quantities of functional groups established on the biochar’s surface are contingent upon factors such as feedstock selection, pyrolysis conditions, and any pre-treatment processes applied. Functional groups on biochar can be classified as acidic, neutral, or basic, with examples including phenolic, carboxyl, lactonic, pyrone, and chromene groups. Chemical modifications of biochar have been shown to enhance its adsorption affinity for various pollutants. For instance, the introduction of oxygen, nitrogen, and sulfur-containing functional groups has been found to significantly improve adsorption potential by altering pore volume and surface area. Notably, the introduction of hydroxyl groups (-OH) can lead to a threefold increase in biochar’s surface area, enhancing its overall adsorption capacity [31]. Furthermore, studies exploring the modification of biochar with nitrogen-containing functional groups have demonstrated improvements in adsorption performance, indicating that these functional groups play a crucial role in enhancing biochar’s ability to capture a range of pollutants [32]. Yinxue synthesized the different types of biochar (CC (corncob biochar), CC600 (corncob biochar pyrolyzed at 600 °C), PB600 (phosphoric-acid-activated biochar at 600 °C), NHB600 (aminated biochar at 600 °C), SBC600 (sulfonated biochar at 600 °C), HAPC600 (hydroxyapatite-loaded biochar at 600 °C), NBC600 (nitrogen-doped biochar at 600 °C), MBC600 (KMnO_4_-oxidized biochar at 600 °C), and HBC600 (H_2_O_2_-oxidized biochar at 600 °C), as shown in Figure 4a–j, and among the several types of functionalized biochar, PB 600 (phosphoric-acid-activated biochar) exhibited the highest adsorption capacity (under acidic and neutral conditions) for sulfamethoxazole (SMX), achieving 195 and 174 mg/g, respectively. The reason for the highest adsorption capacity of PB 600 may be due to the introduction of oxygen containing functional groups (-COOH), which increases the surface functionalities to 211% as compared to the unmodified biochar. This modification enhances the degree of π*-π electron donor–acceptor interactions from 35% before functionalization to 48% after functionalization treatment at pH 2. Furthermore, in a fixed-bed reactor, the PB600 demonstrated strong continuous adsorption performance capability (dynamic adsorption capacity of up to 191 mg/g) at a sulfamethoxazole (SMX) concentration of 50 mg/L. Under alkaline conditions, NHB 600 (amino-modified biochar) also showed superior performance (155 mg/g). It also maintained good performance when applied to real cattle manure effluent, highlighting the effectiveness of the targeted functionalized design of biochar for the removal of SMX antibiotics. Similarly, amination introduced a large amount of -NH_2_ groups to increase surface functional groups to up to 336% of the original level, increasing the adsorption contribution degree contribution of negative-charge-assisted hydrogen bonding from 14% to 37% at pH 10 (result shown as Figure 4k,l) [33]. For cation-exchange capacity (CEC), the property related to CEC indicates biochar’s ability to retain and exchange cations, which is crucial to removing positively charged pollutants [34]. In elemental composition, the ratios of carbon, hydrogen, oxygen, and other elements affect biochar’s stability and reactivity, influencing its adsorption behavior. Biochar’s pH level influences the ionization of functional groups and the availability of charged sites for adsorption [35]. Another important factor influencing the adsorption mechanism of biochar is its hydrophobicity or hydrophilicity. The capacity for adsorption and the interaction between the adsorbate (the pollutant) and adsorbent (biochar) can be quantified using a polarity index. Fresh biochar typically exhibits low oxidation activity, rendering it hydrophobic; this characteristic facilitates the adsorption of organic contaminants primarily through partitioning mechanisms. Most organic pollutants (such as hydrophobic compounds, aromatic dyes, phenols, pharmaceuticals, and other organic micro-pollutants) adhere to biochar’s surface due to hydrophobic interactions, π–π stacking interactions, and pore filling, all of which contribute to the effective removal of these contaminants from water. Hydrophobicity plays a critical role in these processes, as it amplifies the attraction between the organic molecules and the biochar’s matrix. Conversely, various oxidation reactions can increase the hydrophilicity of biochar, thus promoting the adsorption of hydrophilic contaminants. Research has shown that these oxidation processes are often driven by the presence of surface functional groups, which modify biochar’s properties and enhance its interaction with different types of pollutants [36]. Consequently, understanding and manipulating the hydrophobic and hydrophilic characteristics of biochar is essential to optimizing its performance in environmental remediation applications. Such modifications not only enhance the chemical interactions with target contaminants but also contribute to the overall efficacy of biochar as a sustainable solution for environmental remediation.

## 3. Synthesis, Production, and Modification of Biochar

**Figure 5:**
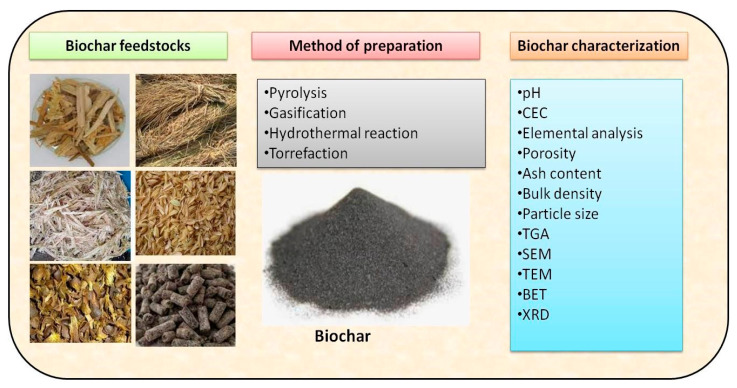
Schematic representation of the biochar feedstock, its preparation process, and characterization techniques.

Biochar has proven to be an effective sorbent for the removal of various inorganic and organic contaminants from contaminated water and soil. The extensive availability of various precursor biomasses ranging from agricultural residues to forestry waste facilitates biochar production, and its relatively simple preparation process enhances its appeal for environmental remediation [37]. Figure 5 provides a brief summary of biochar preparation and characterization. The selected biomass feedstock is first pre-treated through processes (drying, grinding, and sieving) to ensure uniformity prior to pyrolysis under controlled temperature conditions. After carbonization, the resulting biochar is collected, thoroughly washed to remove impurities, and subsequently modified or functionalized depending on the study objective.

**Figure 6:**
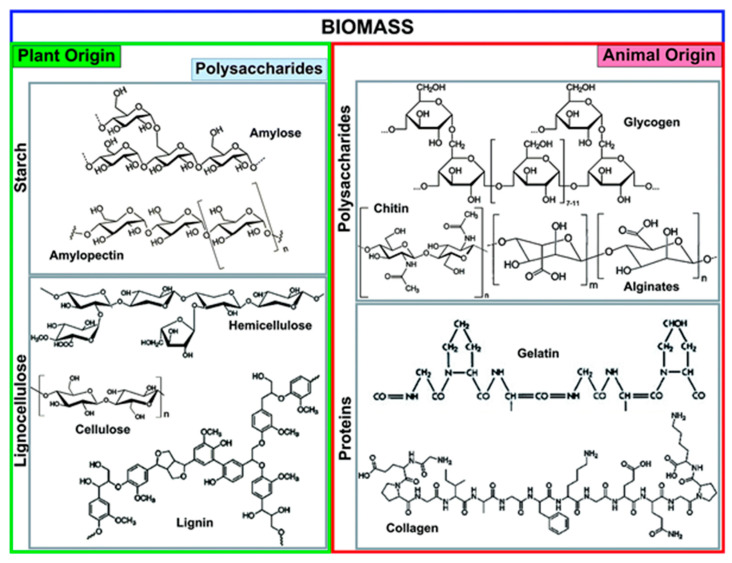
Representative prominent biochemical fractions of biomass feedstocks derived from plants and animals used for biochar production (reproduced with permission, Copyright 2018, *Royal Society of Chemistry*) [38].

Figure 6 illustrates the principal components (biomass feedstocks derived from both plant and animal origins) which serve as precursors for biochar production (by pyrolysis hydrolysis, gasification, or carbonization/calcination at high temperatures). The main categories of biomass include starch, lignocellulose, polysaccharides, and animal-based proteins. Plant-derived biomass primarily consists of starch and lignocellulosic matter. The starch contains glucose units (connected via glycosidic linkage bonds), forming two major molecules (amylose, amylopectin). Lignocellulose composition (made up of cellulose, hemicellulose, and lignin) differs based on the source and depending on its origin (hardwood, softwood, or grasses). Lignin, the second most abundant natural polymer and composed of phenyl propane units, is highly electronegative and may show strong binding affinity toward electropositive metal. Similarly, the animal-derived biomass contains polysaccharides (chitin, glycogen, and alginates) as well as proteins like gelatin and collagen. Chitin (abundant animal-origin polysaccharides) is commonly found in the exoskeletons of insects. The abundance of amino and hydroxyl groups in these biopolymers enhances their affinity for pollutant removal/adsorption (metal ions, dyes) and provides reactive sites for the modification of other functional materials [38]. Similarly, Shi et al. [39] synthesized biochars from wood chips by first oven-drying the material, then grinding it into a fine powder and sieving it to obtain particles in the 200–300 mesh size range. The sieved material was then washed several times with deionized water (D.I) to remove any remaining surface impurities and then oven-dried again. The dried wood powder was mixed with NaOH and then pyrolyzed at 600 °C for 2 h (under continuous nitrogen, flow rate: 20 mL min^−1^, heating rate of 5 °C min). The resulting biochar was repeatedly washed several times with ethanol and D.I water [39]. The ability to recycle waste materials into biochar contributes to sustainability and waste management efforts. However, pristine biochar often exhibits limited adsorption capacity for pollutants due to its low surface area and specific functional groups. To overcome this limitation, researchers globally have explored a range of modification techniques aimed at enhancing the sorption capacity of biochar, making it more effective in addressing water contamination challenges [28,40]. These modification techniques can be broadly categorized into physical and chemical methods, which can occur before, during, or after biochar formation.

**Figure 7:**
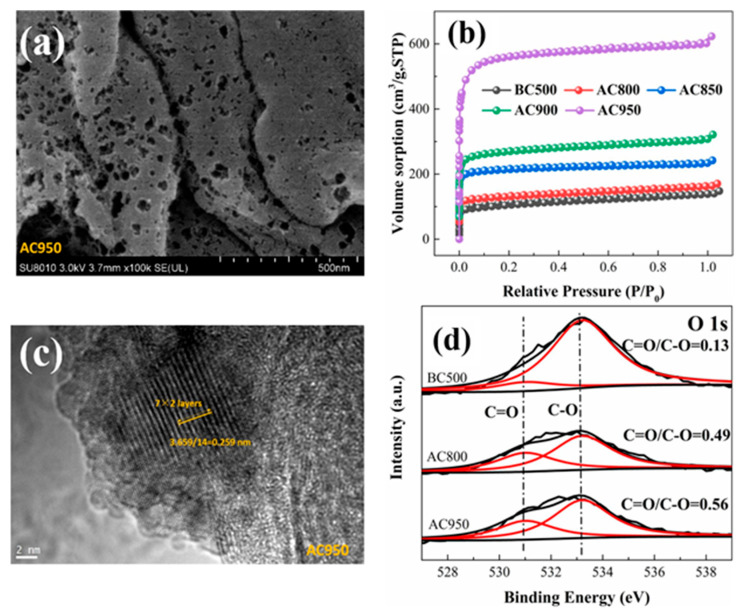
(**a**) SEM image; (**b**) N_2_ adsorption isotherms; (**c**) TEM image; (**d**) high-resolution image of SMX-activated biochars (reproduced with permission, Copyright 2020, *Elsevier*) [41].

There are several modification techniques used for biochar. Common physical modification methods include activation processes, physical blending, chemical activation, and biological modification. Chemical and physical modification of biochar occurs by amplifying its adsorption capacity for specific industrial contaminants. Biochar can be modified using chemical agents such as acids, alkalis, or metal oxides, in addition to physical methods such as steam activation and ball milling. These modifications can significantly increase the surface area, pore structure, and active functional groups of biochar, making it highly effective in treating industrial wastewater. In the physical modification method, pristine biochar is subjected to structured gasification, a process that occurs at elevated temperatures within a controlled environment. This method significantly alters the textural characteristics of biochar, including surface area, pore volume, distribution, and size. For instance, gasification can enhance the porosity of biochars, resulting in a more extensive surface area that facilitates increased adsorption capacity for contaminants. In activation processes, steam or reactive gaseous agents (CO_2_ or O_2_) are typically used to enhance porosity and surface area. This process increases the availability of active sites for adsorption and can be easily scaled up for industrial applications [27]. Sun reported the synthesis of CO_2_-activated biochar (AC) as a catalyst for persulfate (PS) activation, enabling the degradation of phenol and chlorophenol. The precursor cellulose was first pyrolyzed (500 °C, 10 °C/min, 3 h) in a N_2_ atmosphere, and the obtained biochar was activated in a CO_2_ atmosphere (500 mL/min) in a tube furnace for 1 h. SEM/TEM images of CO_2_-activated biochar (AC) are shown in Figure 7a,c. These images show the well-developed microporous structure of CO_2_-activated biochar (AC). The XRD patterns in Figure 7b show peaks at 26.4° (002) and 44° (100), which represent the diffraction of graphite (partial graphitization during the activation process). This activation of biochar is beneficial for PS activation via a non-radical mechanism by enhancing the ability of electron transfer. The N_2_ sorption curves indicated that the surface area of the AC increased considerably (506 m^2^/g to 2185 m^2^/g) with a predominantly microporous structure as shown in Figure 7d. The XPS analysis revealed that during CO_2_ activation, the ratio of carbonyl to hydroxyl and epoxide groups increased (0.13 to 0.56), indicating a transformation of surface oxygen functionalities. This change, along with the reduction in overall oxygen content (17.4% to 7.8%), enhanced the biochar’s surface reducibility and facilitation [41]. The physical blending process combines biochar (clays or zeolites) to create composite adsorbents that leverage the strengths of both materials [42,43]. Additionally, this modification process impacts the surface chemical features of biochars, including its polarity, hydrophobicity, and the presence of various functional groups [44]. Changes in polarity and hydrophobicity can influence the interactions between biochar and different types of pollutants, thereby affecting the overall adsorption efficiency. For example, enhanced hydrophobicity may improve the sorption of organic contaminants, while increased polarity can facilitate the adsorption of hydrophilic substances [44,45]. The generation of specific surface functional groups during gasification can further tailor biochar for targeted applications, as these groups can enhance chemical bonding with contaminants including heavy metals (Pb^2+^, Cd^2+^, Cu^2+^) and organic pollutants (dyes, phenols, and pharmaceuticals). Overall, physical modification through structured gasification not only optimizes the structural and chemical properties of biochars but also improves its effectiveness as a sorbent in environmental remediation efforts.

**Figure 8:**
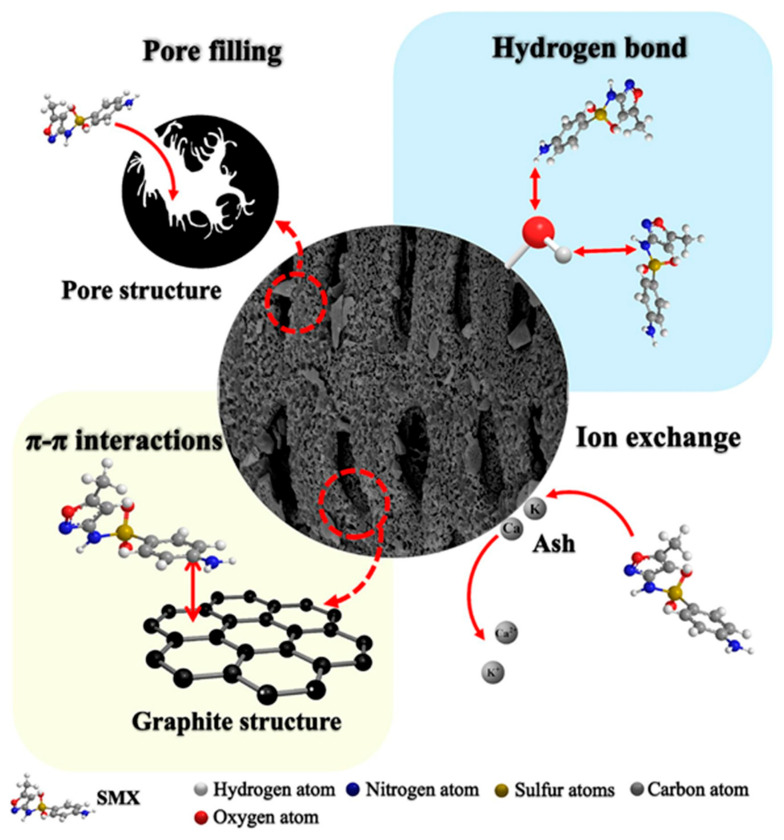
Proposed adsorption mechanisms of biochars for pollutant removal (reproduced with permission, Copyright 2025, *Elsevier*) [53].

Steam activation is one of the most commonly employed methods for modifying biochar, utilizing water vapor as the oxidizing agent [46]. This technique is particularly effective for enhancing the structural properties of biochars and for removing byproducts generated during incomplete combustion processes. Steam activation increases the surface area available for the sorption of adsorbates, thereby improving the material adsorption capacity [47,48]. This process specifically targets the removal of volatile carbon from pristine biochar, leading to the creation of porosity and an increase in specific surface area. Several factors influence the effectiveness of steam activation, including the rate of steam supply, and the nature of the precursor biomass and its chemical composition [49]. Typically, steam-activated biochar exhibits excellent micro-porosity, while contributing minimal meso-porosity. This characteristic is due to steam activation primarily promoting the development of carbon microspores [50]. Additionally, the process involves the oxidation of biochar materials and the production of syngas, particularly hydrogen, which further enhances the surface area of the resulting biochar [51]. For example, Xie et al. [52] reported that the surface area of biochar derived from silk can be increased by 122 to 197 times after steam activation. This significant enhancement underscores the potential of steam activation as an effective method for optimizing biochar properties for various environmental applications [52]. Recently, bamboo-activated carbon manufactured by water steam activation at different temperatures and used for adsorption of sulfamethoxazole was reported. Figure 8 illustrates proposed biochar adsorption mechanisms for pollutant removal and demonstrates the key adsorption-interaction mechanisms of steam-activated biochar, showing the pore filling, H-bonding, ion exchange, and π–π interactions. Pore filling was a stable mechanism for sulfamethoxazole removal (improved by abundant pore structure). The steam-activated biochar increased in specific surface area (56.97–87.73 times), with an adsorption capacity of 204.07 mg/g (significantly influencing pore-filling adsorption capacity). These combined processes enhanced the adsorption capacity by making effective use of its porous structure, active surface functional groups, and mineral constituents [53].

**Figure 9:**
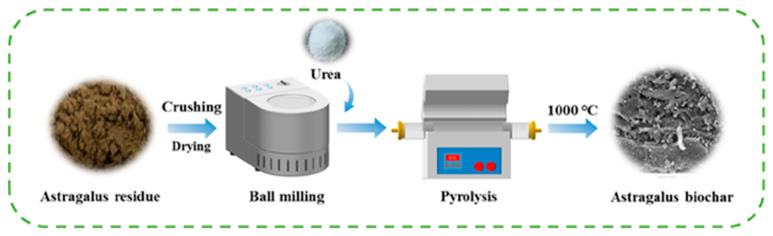
Preparation flow chart of biochars from Astragalus (*Astragali radix*) by ball milling method (reproduced with permission, Copyright 2025, *Elsevier*) [56].

Ball milling, often referred to as solid phase synthesis, is an established technique for creating nano-sized materials and is widely utilized in industrial applications. In biochar modification, ball milling is increasingly recognized as an effective approach to produce biochar-based adsorbents with enhanced properties. A preparation flow chart of biochar using the ball milling method is provided in Figure 9. Materials synthesized using this method exhibit high porosity and good dispersion, significantly improving their efficiency in treating environmental contaminants. This innovative and cost-effective technology allows for the creation of carbon-based composites with superior surface characteristics, enhancing the biochar’s adsorption capabilities [54,55,56]. The physicochemical properties of biochar and biochar nanocomposites, including microporous surface area, porosity, pore volume, and pore distribution, are all improved through ball milling. These enhancements contribute to a marked increase in the adsorption capacity of biochar, enabling it to effectively capture both organic and inorganic contaminants from polluted environments. Research indicates that the efficacy of biochar can be enhanced up to 200-fold following ball milling compared to unmodified biochar materials. For example, Li et al. [57] demonstrated that ball-milled biochar can effectively remediate pharmaceutical compounds and heavy metal ions from a synthetically prepared solution. Specifically, tetracycline (TC) and mercury (Hg) were successfully removed from contaminated water using magnetic ball-milled nano-biochars, achieving an impressive removal efficiency of approximately 99% [57]. Additionally, a study by Zhang et al. [58] highlighted the ability of ball-milled biochar to efficiently remove organic pollutants, such as methylene blue, from water. In their research, hickory chips were used to prepare biochar, which was then modified through ball milling, followed by treatment with a 10% hydrogen peroxide (H_2_O_2_) solution. This dual modification significantly enhanced the surface properties of the biochar, increasing the number of functional groups, such as hydroxyl and carboxyl groups. As a result, the modified biochar demonstrated effective removal of methylene blue from synthetically prepared solutions [58].

Chemical modification methods often include the: (a) acidic or alkaline treatments: These methods modify the surface chemistry of biochar, introducing functional groups that improve adsorption capacity for specific contaminants; (b) loading of nanomaterials: Incorporating nanoparticles (iron oxides or carbon nanotubes) enhances the reactivity and adsorption capabilities of biochars, particularly for heavy metals and organic pollutants [59,60,61]. While chemical modifications can significantly enhance a biochar’s performance, they may require costly chemicals and can sometimes lead to secondary environmental concerns due to potential pollution [62,63]. Chemical modification is a prominent approach for enhancing the properties of biochars, employing various agents such as alkaline and acidic solutions, oxidizing agents, and metal oxides or salts. These chemical treatments effectively alter the surface characteristics of biochar, including the creation of surface defects and the modification of the types and concentrations of functional groups [64,65]. By applying acids (sulfuric or hydrochloric acid), the introduction of acidic functional groups can improve the biochars’ ability to interact with pollutants. Conversely, alkaline treatments with sodium hydroxide or potassium hydroxide can enhance the porosity and surface area of biochar, promoting the adsorption of contaminants. Oxidizing agents, such as hydrogen peroxide or ozone, can also be employed to increase the number of oxygen-containing functional groups on the biochar surface. This modification can enhance its hydrophilicity and improve the adsorption capacity for a wider range of pollutants, including both organic and inorganic substances [39,65]. Additionally, the incorporation of metal oxides or salts during the modification process can create composites that leverage the properties of both biochars and the added materials. These modifications not only improve the adsorption efficiency of the biochar but also enhance its structural stability and reactivity [66]. Acid modification is an effective method for enhancing the surface characteristics of biochar, particularly for the removal of metals and other contaminants. This treatment introduces a significant number of new functional groups, which can improve biochar’s adsorption capacity. According to Wang and Wang [67], the specific surface area of biochars can be modified by acid treatment, with the effects varying depending on the concentration and type of acid used. Acidified biochar has been identified as an excellent soil enhancer. Studies have shown that the addition of acids during the hydrothermal carbonization of biomass can significantly increase the adsorptive capacity of hydro-char [67]. For example, Reza et al. (2015) reported that the incorporation of acetic acid into wheat straw hydro-char during hydrothermal carbonization resulted in enhanced surface area and porosity [68]. Furthermore, He et al. [69] produced biochar from rice straw under oxygen-deficient conditions and subsequently modified it with a 15% solution of hydrogen peroxide (H_2_O_2_) and a mixture of nitric (HNO_3_) and sulfuric (H_2_SO_4_) acids in a 1:1 ratio. This acid modification led to a substantial increase in carboxylic functional groups (-COOH) on the biochar surface [69]. Notably, acid modifications were found to have a more pronounced effect on the biochar’s functional group composition than oxidation treatments. Ultimately, acid treatment not only alters the functional groups and surface charge of biochar but also enhances the purity of its surface by decreasing the point of zero charge. These modifications are essential for optimizing the properties of biochar materials for various applications, particularly in environmental remediation [28,40].

The primary aim of alkali modification is to enhance the number of oxygen-containing surface functional groups present on biochar materials. Common alkaline chemicals used for this modification include 1M sodium hydroxide (NaOH), potassium hydroxide (KOH), and potassium carbonate (K_2_CO_3_) [70]. These alkaline agents are effective in augmenting the specific properties of biochar, influencing its surface area and adsorption capabilities. The type of feedstock and the conditions under which biochar is produced also significantly affect its surface characteristics, which can be further modified through alkaline treatments [52]. For instance, Enaime et al. [71] prepared biochar from solid olive waste and activated it using KOH to treat effluent from an olive mill. The KOH-treated biochar exhibited impressive microporous volume and specific surface area, measuring 0.52 cm^3^/g and 1375 m^2^/g, respectively. This enhanced structure resulted in a high adsorption efficiency for indigo carmine, achieving a capacity of 599 mg/g. In another study, KOH was used to modify biochar derived from municipal waste, resulting in an increase in specific surface area up to 49.1 m^2^/g. This modification also improved the biochar’s capacity to remove arsenate (As(V)) effectively [71]. Furthermore, Tang et al. [72] developed an alkali-modified biochar using straw to eliminate emerging contaminants from kitchen waste, including bisphenol A (BPA) and antibiotics such as ofloxacin (OFL) and tetracycline (TC). The modified biochar demonstrated removal efficiencies ranging from 95% to 100%, with sorption capacities of 71.4 mg/g for BPA, 101.0 mg/g for TC, and 54.0 mg/g for OFL. The straw biochar treated with alkaline chemicals exhibited increased hydrophobicity, enhanced surface area, and improved adsorption capabilities, making it highly effective for removing a range of contaminants [72].

**Figure 10:**
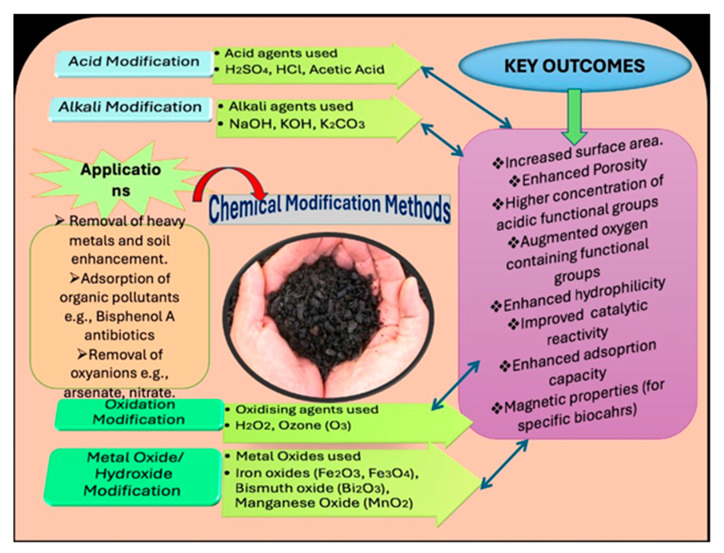
Chemical modification of biochar: the chemical activation and surface modification steps applied to raw biochar.

In recent years, the production of metal oxides and salts for modifying biochars has gained significant attention. This modification technique enhances the core characteristics of biochar, improving its adsorption capacity, catalytic activity, and magnetic properties [66]. Metal oxide modifications increase biochar ability to bind inorganic and organic contaminants by enhancing ion exchange and functional group interactions. Typically, biochar materials possess a high surface area, elevated pH, and negatively charged surfaces, making them excellent adsorbents for metal cations through mechanisms like precipitation and electrostatic interactions. However, pristine biochar struggles to effectively adsorb oxyanions such as nitrate, arsenic, and phosphate. To address this limitation, metal oxide modification has emerged as a powerful tool. For instance, Amen et al. [22] reported bismuth-modified biochar using rice husk as the precursor. The precursor was pretreated with a solution of bismuth oxide and carbonized at various temperatures ranging from 400 °C to 600 °C. The biochar synthesized at 500 °C demonstrated the highest adsorption capacity for arsenic removal from contaminated water. This biochar exhibited a higher pore volume, surface area and a maximum adsorption capacity (q_max_) of around 16 mg/g at a slightly alkaline pH of 9.2. Iron-impregnated biochar, which possesses magnetic properties, has also been synthesized using waste from timber tree leaves, vegetable leaves, and fruit waste. This magnetic biochar showed remarkable efficiency in removing both arsenite (As(III)) and arsenate (As(V)) from contaminated water [73,74,75,76]. Additionally, manganese-modified biochar has been developed by infusing manganese oxide into Tectona leaf waste. The waste was carbonized under anaerobic conditions at 800 °C for one hour and then applied to remove trivalent arsenic (As(III)) from a synthetically prepared solution [75]. Overall, chemical methods provide versatile and effective means to tailor the surface properties of biochar, making it a more powerful tool for environmental remediation and pollutant removal (Figure 10).

**Figure 11:**
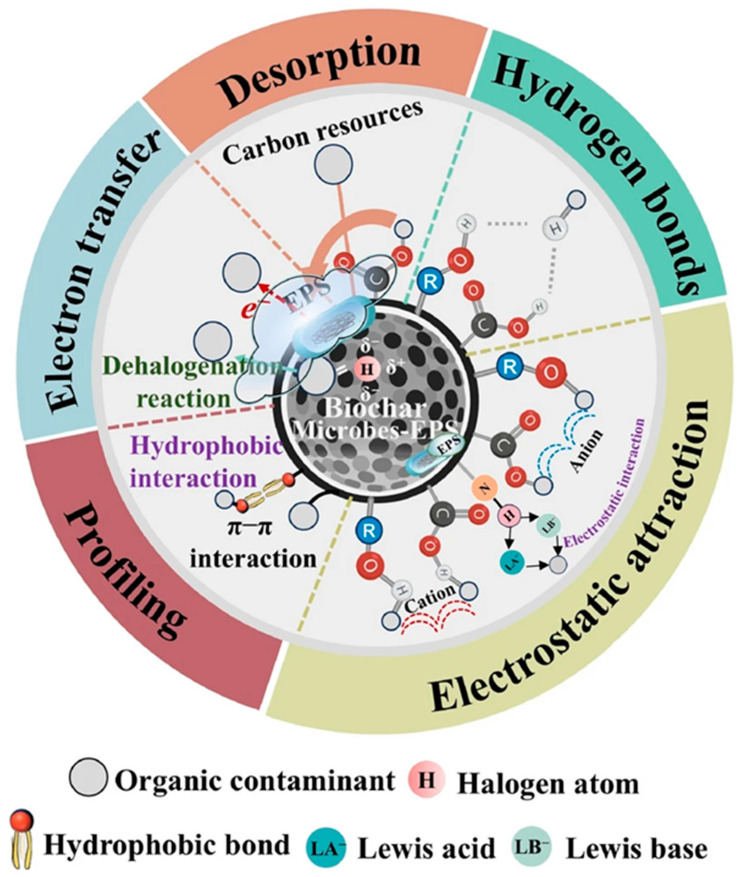
The microbial-EPS (Extracellular Polymeric Substance)-biochar systems (which stabilize heavy metals by electrostatic attraction, H-bonding, ion exchange, and complex formation), mediated heavy metal bioremediation mechanisms which effectively lower mobility and potential toxicity (reproduced with permission, Copyright 2025, *Springer Nature*) [79].

Biological modification, which employs microbial processes to alter the properties of biochar, can also be effective. While scalable, this method often requires longer contact times, which may alter the surface and structural characteristics of the biochar [77]. The biological method has been particularly effective in treating wastewater with low contamination levels. The use of microbes to adsorb both organic and inorganic contaminants from polluted water has gained significant attention due to its promising, inexpensive, simple, and efficient nature. Microorganisms such as yeast, fungi, algae, and bacteria found in industrial residual biomass have been shown to effectively accumulate heavy metals. These microbes form biofilms that exhibit a variety of functional sites, including hydroxyl, carbonyl, carboxyl, and amino groups. Enhancing the adsorption potential of biochar materials can be achieved by surface grafting and functional group exchange. Biochar, an inert material with a large surface area, supports the establishment of biofilms with favorable properties, facilitating the biological breakdown of organic pollutants. This is often done by inoculating microbes onto the biochar surface to accelerate the degradation process. Chen et al. [78] demonstrated the effectiveness of this method by using sewage sludge (SS) and rice husk (RH) to prepare alkaline biochar. The biochar was then modified with phosphate-solubilizing bacteria (PSB) and applied to reduce lead (Pb(II)) in contaminated water. The results showed that Pb(II) removal efficiencies were 24.11% and 60.85% for RHPSB and SSPSB, respectively, highlighting the significant potential of biologically modified biochar in environmental remediation [78]. Figure 11 shows the microbial-EPS-biochar-mediated mechanisms of heavy metal bioremediation, illustrating how the strong surface charge of biochar limits the mobility and environmental impact of heavy metals. It promotes complex formation and electrostatic attraction. This complex formation decreases the bioavailability, ecological impact, and leaching potential of heavy metals in depositional environments. The oxygen-containing functional groups (present on the biochar surface) facilitate the metal deposition and adsorption (by ion exchange with metal cations). The ionized functional groups serve as active binding sites and form organometallic complexes by electrostatic interactions (by lowering the toxicity of heavy metals to microorganisms) [79]. The use of biological methods to modify biochar not only enhances its adsorption capacity but also supports sustainable environmental practices by reducing the need for chemical agents. Microbial-assisted biochar modification focuses on harnessing the natural capabilities of various microorganisms to degrade organic pollutants and immobilize inorganic contaminants. These microbes can utilize the biochar surface as a habitat, where they thrive and perform essential metabolic activities that aid in the bioremediation process [79,80]. There are several advantages of biological modification: (a) cost-effectiveness: Since the microbes used in the process often come from residual biomass of industries, the method is cost-efficient and can be scaled for large applications without the need for expensive chemicals. (b) Eco-friendly approach: Biological methods avoid the use of hazardous chemicals, thereby reducing secondary pollution during the treatment process. This makes it a more environmentally friendly approach to wastewater treatment and soil remediation. (c) Enhanced bioavailability of nutrients: Certain microbes, such as phosphate-solubilizing bacteria (PSB), can improve the bioavailability of essential nutrients like phosphates. This can have a dual benefit of pollutant removal and soil fertility enhancement, particularly in agricultural applications. (d) Synergistic effects: In many cases, the biochar provides a stable matrix and a large surface area for microbial colonization, creating a synergistic effect where biochar supports microbial activity while the microbes enhance biochar’s adsorption potential for contaminants [81,82]. Key microbial mechanisms include biosorption, where microorganisms have functional groups on their cell walls, such as hydroxyl and carboxyl groups, that can bind with heavy metals, enabling effective adsorption of contaminants like lead, cadmium, mercury, and arsenic; and biodegradation, where microbes can break down organic pollutants like hydrocarbons, dyes, and pharmaceuticals into simpler, non-toxic compounds through enzymatic action. This biodegradation process can be enhanced when microbes are supported by biochar’s porous structure [83,84]. Biofilm formation: Biofilms formed by microbes on biochar surfaces act as an efficient system for pollutant degradation and adsorption. Biofilms provide a protective environment for microorganisms, enhancing their longevity and activity, which contributes to the long-term sustainability of biochar-based water-treatment systems. Biochar presents a viable option for the removal of contaminants; its effectiveness can be significantly improved through tailored modification techniques that enhance its adsorption capacity and adapt it for specific applications. Ongoing research into optimizing these modifications will be crucial for developing sustainable and effective biochar-based solutions for environmental remediation.

## 4. Biochar Applications and Performance

**Figure 12:**
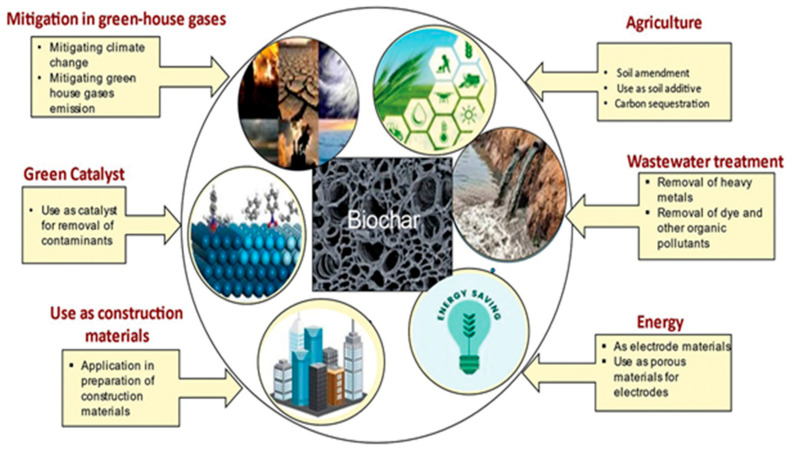
Different applications of biochar (reproduced with permission, Copyright 2024, *American Chemical Society*) [93].

Biochar is used in various environmental, agricultural, and industrial applications owing to its large surface area, porous structure, and abundance of active functional groups as shown in Figure 12. Heavy metal removal: Microbial biochar has proven to be effective for removing a wide range of heavy metals, including lead (Pb), cadmium (Cd), and arsenic (As), from both water and soil. In treating industrial wastewater, biochar removes contaminants mainly through adsorption, redox processes, and catalytic degradation. Heavy metal adsorption: The negatively charged surface and abundant oxygen-containing functional groups (such as carboxyl, hydroxyl, and carbonyl) on biochar interact with positively charged metal ions (Cu^2+^, Pb^2+^, and Ni^2+^) through electrostatic interactions, ion exchange, and surface complexation, effectively reducing metal concentrations in wastewater [85,86]. Organic pollutant degradation: The biological modification of biochar has shown significant results in degrading pharmaceutical compounds, pesticides, and industrial dyes, making it an ideal solution for wastewater treatment [87,88]. Organic pollutants, including dyes, phenols, and hydrocarbons, can be adsorbed onto biochar surfaces via hydrophobic interactions, π–π interactions, and van der Waals forces. Biochar’s ability to adsorb organic compounds aids in treating effluents from industries like textile, chemical manufacturing, and petroleum refineries [87,88,89]. Dye and textile industry: The adsorption capacity of biochar for organic dyes, such as methylene blue and indigo carmine, makes it a sustainable alternative for dye-laden wastewater treatment [90]. Enhanced soil remediation: Biochar modified with nitrogen-fixing bacteria or phosphate-solubilizing bacteria can also improve soil quality by aiding nutrient cycling, making this method beneficial for both environmental and agricultural applications [91]. Chemical and petrochemical industry: Biochar can be used to remove organic pollutants and heavy metals from effluents produced by chemical manufacturing, petroleum refining, and other related industries. Battery production: Wastewater generated by battery production processes contains high levels of metals such as cadmium, nickel, and lead. Wang et al. [92] found that biochar-supported microbial biofilms significantly improved the sorption and degradation of organic pollutants due to the high density of functional sites provided by the biofilm. The versatility of this approach allows it to be used in various environmental applications, making it a promising method for large-scale pollution control [92]. Biochar has gained attention as a promising technology for negative carbon emissions and has also shown significant potential in wastewater treatment. Its porous structure, high surface area, and abundance of functional groups make biochar an excellent material for adsorption-based pollutant removal. Biochar can be employed in various ways to treat wastewater, including as an economical biosorbent to remove contaminants like heavy metals, organic pollutants, and excess nutrients. Additionally, biochar can enhance water quality by improving soil conditions, which is vital in preventing runoff pollution [91,92]. Mining and metal processing: Biochar is particularly effective in adsorbing heavy metals such as arsenic, lead, and mercury from wastewater streams generated by mining and metal processing industries.

**Figure 13:**
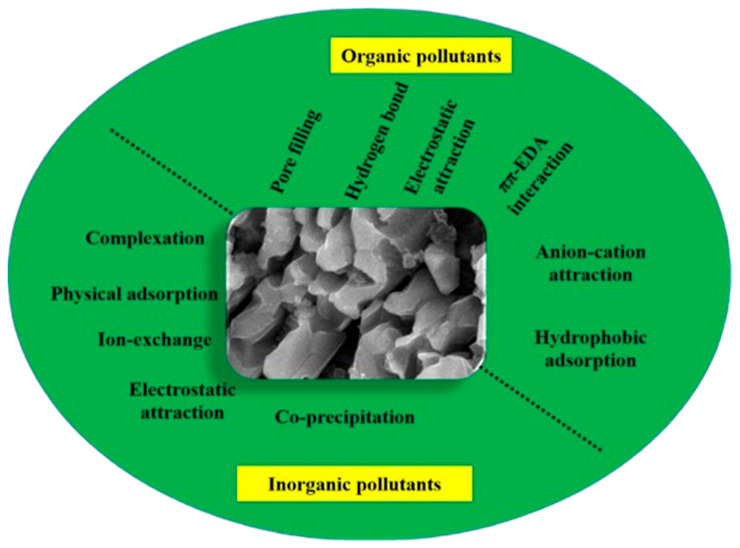
Possible mechanism of inorganic and organic contaminant adsorption by the use of biochar.

The mechanism of biochar for pollutant removal involves various pathways, influenced by the nature of both the biochar and the pollutants. Understanding these mechanisms is essential to optimizing biochar efficiency in removing contaminants from wastewater. Figure 13 illustrates these possible mechanisms for the removal of both organic and inorganic pollutants [27,80]. The possible mechanisms of biochar for pollutant removal occur through the following routes: adsorption routes, organic pollutant removal mechanisms, and inorganic pollutant removal mechanisms. (a) The adsorption route includes physical adsorption, pore filling, and precipitation: Physical adsorption involves weak interactions between the biochar surface and the adsorbate, where contaminants are attached to the surface without undergoing any chemical transformation [94]. In pore filling, the contaminants can condense and get trapped within the pores of biochar. This depends on the structure of the biochar, such as if it is mesoporous and microspores, which influences its adsorption capacity [57,95]. In precipitation, the pollutants form a layer over the biochar surface through chemical processes such as precipitation or crystallization [96]. (b) Organic pollutant removal mechanisms involve hydrophobic interaction, electrostatic attraction, and hydrogen bond formation. In hydrophobic interaction, organic contaminants, particularly those with hydrophobic properties, can be adsorbed on the hydrophobic biochar surface, which often contains hydrophobic functional groups [71]. Electrostatic-attraction biochar, which usually carries a negative charge, can attract positively charged organic pollutants through electrostatic forces. This mechanism becomes prominent for polar organic contaminants, particularly when biochar loses oxygenated functional groups at higher pyrolysis temperatures [97]. Hydrogen-bond formation electrostatic repulsion between biochar and negatively charged organic pollutants may also facilitate hydrogen bonding, which enhances the adsorption of organic ions [72,98]. (c) Inorganic pollutant removal mechanisms involve surface complexation, electrostatic interaction, and precipitation. In surface complexation, oxygenated functional groups, such as carboxyl (-COOH) and hydroxyl (-OH) groups, on biochar can interact with inorganic contaminants like heavy metals via surface complexation, forming stable complexes [73,75]. In electrostatic interaction, similar to organic pollutants, inorganic contaminants with positive charges, such as metal cations, are attracted to the negatively charged biochar surface through electrostatic forces [57,95]. In precipitation, the heavy metals can precipitate on the biochar surface under certain chemical conditions, contributing to their removal. The specific mechanism of adsorption is influenced by the functional groups present on the biochar surface. Higher pyrolysis temperatures reduce oxygenated and hydrogenated functional groups, making the biochar more aromatic and less polar, thus altering its effectiveness in removing polar contaminants [99]. Inorganic contaminants such as heavy metals interact with biochar mainly through oxygen-containing groups, while changes in biochar surface chemistry before and after adsorption (measured via FTIR) reveal shifts in peaks and bonding intensities, revealing the nature of adsorption interactions. These diverse mechanisms make biochar a highly adaptable material for the treatment of various pollutants in wastewater. The adsorption capacity and efficiency can be tailored by modifying the biochar physical structure and surface chemistry, allowing for the selective removal of different contaminants.

Biochar purifies wastewater primarily through adsorption, ion exchange, and surface complexation mechanisms. Its highly porous structure and abundant functional groups promote strong interactions with contaminants. Adsorption: Biochar’s porous structure and high surface area allow it to adsorb organic and inorganic contaminants effectively. Ion exchange: Functional groups present on biochar surfaces, such as carboxyl and hydroxyl groups, enable ion exchange processes that bind pollutants like heavy metals. Catalytic degradation: Biochar modified with metals or nanomaterials can catalyze the breakdown of certain organic pollutants in water. Synergistic effect with microbes: Biochar can serve as a support for microbial communities, enhancing the degradation of pollutants through biological processes [100,101,102]. Table 1 and Table 2 present the removal efficiencies and adsorption capacities of biochar for various contaminants. The table highlights biochar versatility and potential in addressing diverse wastewater pollutants. With advances in biochar modification techniques, its effectiveness continues to improve, making it an attractive option for sustainable water treatment solutions.

Industries are one of the leading sources of wastewater pollution, contributing to the degradation of water quality through the release of both organic and inorganic contaminants. Biochar has emerged as a promising and effective solution for the removal of these industrial contaminants. Its high surface area, porosity, and functional groups make biochar a suitable adsorbent for trapping and immobilizing heavy metals and other toxic compounds present in industrial effluent [89]. The concentration of industrial pollutants has been rapidly increasing due to diverse industrial activities, including battery production, mining, smelting, leather tanning, dye manufacturing, and chemical production [104]. Among the pollutants, heavy metals (arsenic (As), copper (Cu), lead (Pb), chromium (Cr), nickel (Ni), and mercury (Hg)) are of particular concern due to their toxicity and persistence in the environment [105]. Biochar has garnered considerable attention for its potential to remove organic pollutants like dyes, phenols, and heavy metals from industrial wastewater. For instance, Cr(VI) adsorption using biochar derived from glue residue modified with ZnCl_2_ achieved a remarkable adsorption capacity of 325.5 mg/g, the highest reported for this contaminant [106]. This highlights biochar’s exceptional capacity to treat heavy metals. In another study, Gayathri et al. [107] developed biochar from agricultural waste (jujube seeds) and successfully used it for the removal of lead and zinc from electroplating industry wastewater. The biochar was first modified with H_2_SO_4_ and then subjected to ultrasonic wave treatment, which significantly enhanced its adsorption capacity. The biochar achieved an adsorption capacity of 119.8 mg/g for zinc and 221.1 mg/g for lead. This modified biochar was also effective in removing other metal ions like nickel, copper, and chromium, commonly generated in electroplating processes [107]. Additionally, acid-modified biochar synthesized from corncob was utilized to treat dye-laden wastewater from the textile industry. The corncob biochar was pyrolyzed at 450–550 °C and impregnated with sulfuric acid to enhance its adsorption properties. This acid-treated biochar exhibited an impressive decolorization efficiency of 98%, with the highest dye adsorption capacity reaching 6.02 mg/g in just 45 min at room temperature [108]. Li et al. [95] demonstrated the removal of Cr from wastewater using Mg-impregnated biochar. The study revealed that the Cr was removed through a combination of electrostatic attraction, bonding with functional groups, and complexation, highlighting the multifunctional role of biochar in adsorbing inorganic contaminants [95]. These examples underline biochar versatility and effectiveness in industrial wastewater treatment, especially for both organic pollutants and heavy metals. Biochar has been found effective in removing these metal ions from industrial discharge streams. Overall, the use of biochar for industrial wastewater treatment (Table 2) is a promising, cost-effective, and environmentally sustainable approach for mitigating pollution and reducing the harmful impacts of industrial activities on the environment.

**Table 2:** Biochar and its application of the removal of various organic and inorganic pollutants from wastewater.

Biomass for Biochar	Pre/Post-Treatment	Pyrolysis Temperature (°C)	Contaminants	Initial Concentration (mg/L)	Biochar Dose (g/L)	Adsorption Capacity (mg/g)	Mechanism	References
	**Inorganic Contaminants**							
**Banana straw**	MgCl_2_	430	Cr(VI)	50	0.02	125	Complexation, electrostatic attraction, functional group bonding	[108]
**Eucalyptus leaves**	KOH	200	Pb^2+^	10	50	-	Co-precipitation, complexation	[109]
**Cornstalk**	Fe^2+^/ZnCl_2_	700	Pb^2+^	10	10	99.82		
**Coconut shell**	MgCl_2_	400	Cd	300	0.5	81.7	Ion-exchange, metal-π electron coordination, mineral precipitation, and interaction with oxygenated functional groups	[110]
**Coconut shell**	Straw	Alkali treatment	Pb^2+^	300	0.5	214.4		
**Date palm**	NA	800	Pb^2+^	50–250	1	98.9	NA	[111]
	Hickory Chips	Ball-milling, H_2_O_2_ treatment	Cu^2+^	50–250	1	41		
**Corncob**	NA	550	Pb^2+^	1.95 mg/mL	4	NA	Ion diffusion	[22]
			Cd^2+^	0.95 mg/mL	4	NA		
**Brown algae**		300	Zn^2+^	1–180	0.2	1.78	NA	[112]
	**Organic Contaminants**							
**Rice husk**	Peroxymonosulfate	450	Tetracycline	20–100	0.05–0.5	NA	NA	[72]
			Bisphenol A	20–100	0.05–0.5	NA	NA	
**Date palm petiole**	NA	700	Crystal violet	5–500	0.05	209	Pore filling, π–π interaction and H-bonding	[113]
**Corncob**	NA	500	Brilliant green dye	50–200	2	39.4	NA	[114]
**Egg shell**	HCl	800	Rhodamine B	NA	1	NA	NA	[40]
**Cow dung**	KOH/NaOH	700	Rhodamine B	100–300	1	1241	π–π interaction, electrostatic attraction, H-bonding	[115]

Agricultural wastewater, due to the extensive use of fertilizers, pesticides, herbicides, and other chemicals, is becoming a major environmental concern. The release of hazardous substances into farmlands has posed serious threats to human health, food safety, and biodiversity. Heavy metals, pesticides, and other toxic chemicals from agricultural runoff can accumulate in soil and water, leading to long-term contamination of ecosystems and exposure risks to humans and animals [116]. Biochar has emerged as a promising solution for the remediation of agricultural wastewater. Both pristine and modified biochar have been proven to effectively adsorb and remove various pollutants. Biochar’s porous structure, large surface area, and potential for chemical modification make it an ideal adsorbent for reducing the toxicity of heavy metals, pesticides, and other contaminants. Studies show that biochar can enhance soil fertility while also helping to immobilize pollutants, preventing them from leaching into groundwater or being absorbed by crops. The excessive use of chemicals in agriculture, although beneficial for crop yields and the global economy, can severely disrupt ecological balance, harm non-target species, and pose significant health risks to humans [117]. The application of biochar to treat agricultural wastewater can mitigate these issues by serving as a filter for contaminants, promoting sustainable farming practices while protecting the environment from chemical overuse.

Municipal wastewater management has become a critical issue due to rapid urbanization and population growth. The large volume of wastewater generated by households and businesses poses significant environmental challenges, particularly in terms of nutrient pollution. Nitrogen (N) and phosphorous (P) are two of the most prevalent contaminants in municipal wastewater and their excessive presence can lead to eutrophication, a process that results in the excessive growth of algae, which depletes oxygen levels in aquatic environments, endangering aquatic life [118]. Recent studies have shown that biochar can be highly effective in the treatment of municipal wastewater, either used alone or in combination with other advanced treatment technologies. For example, biochar can play a crucial role in the recovery and recycling of phosphorous and nitrogen from wastewater. These nutrients are essential for agricultural use but can cause significant harm to aquatic ecosystems if they reach water bodies in large quantities [119]. The release of phosphorous from both municipal and industrial wastewater is one of the primary human-induced contributors to water pollution, along with agricultural runoff from the use of chemical fertilizers [120]. One promising method involves the impregnation of biochar with aluminum oxyhydroxide (AlOOH), which has been found to efficiently adsorb phosphorous from wastewater through electrostatic attraction. This biochar, once used to capture phosphorous, can be further applied as a slow-release fertilizer, providing a sustainable solution for recycling nutrients [121]. Moreover, innovative technologies combining biochar with membrane filtration systems have been developed. For instance, Zhang et al. [122] designed a municipal wastewater treatment system using an anaerobic membrane bioreactor with biochar as an adsorbent, coupled with reverse osmosis. This integrated system showed improved recovery of valuable nutrients while also effectively treating wastewater. These approaches highlight the potential of biochar to not only reduce the environmental impact of municipal wastewater but also recycle nutrients back into agricultural systems, closing the loop on nutrient management and supporting sustainable water treatment practices [122]. However, it should be noted that the impact of biochar can vary depending on several factors (such as pyrolysis conditions, feedstock type, and application method).

## 5. Future Prospective and Directions

The use of biochar in wastewater treatment presents a promising avenue for developing sustainable solutions to environmental challenges. Its effectiveness in removing pollutants, coupled with its low carbon footprint, positions biochar as a viable alternative for sustainable wastewater management. Here are some key points regarding the future potential of biochar in this field:

➢ Biochar’s ability to adsorb various contaminants from polluted water enhances water quality and makes it a desirable option for wastewater treatment. As awareness of environmental sustainability grows, biochar can serve as an innovative solution that aligns with the principles of a circular economy by conserving resources and minimizing waste.
➢ Beyond its role in wastewater treatment, biochar contributes to climate change mitigation by reducing greenhouse gas (GHG) emissions. Its production from organic waste and subsequent use in soil can sequester carbon, helping to combat climate change while improving soil health.
➢ Biochar is a renewable resource that fits well within the framework of a circular economy. By recycling organic waste into valuable biochar, it not only provides an alternative to waste disposal but also enhances resource efficiency. This approach can lead to significant improvements in resource conservation over extended periods.
➢ Continued research is essential to maximize the potential of biochar in wastewater treatment. Specifically, the following areas require further exploration. Feedstock selection: Different types of biomass yield biochar with varying properties. Investigating the best feedstock for specific wastewater applications can optimize performance. Synthesis conditions: The techniques and parameters used in biochar production such as temperature, time, and chemical treatments can significantly affect its adsorption capacity. Researching these variables will help produce biochar tailored for specific contaminants. Adsorption mechanisms: While chemically treated biochar has shown improved contaminant adsorption compared to unmodified biochar, more in-depth studies are needed to understand the underlying adsorption mechanisms better.
➢ Modification techniques: Various modification strategies, including chemical, physical, and biological methods, have been discussed, showcasing how these enhancements can improve biochar porosity, surface area, and functional groups. Such modifications lead to increased adsorptive performance, making biochar a more viable option for wastewater treatment.
➢ Adsorption mechanisms: The review emphasizes the critical processes involved in biochar adsorption capabilities, elucidating how its structure and surface properties contribute to its efficiency in capturing contaminants.
➢ Diverse applications: The properties of biochar and its production methods, alongside innovative modifications, position it as a multifunctional material that can address both organic and hazardous inorganic pollutants effectively.
➢ Research on the interactions between processing variables, feedstock choices, and the regeneration and disposal of biochar is crucial to mitigate any environmental risks. Understanding how to safely manage biochar waste and its potential effects on soil and water systems will be critical as its use becomes more widespread.
➢ Biochar holds significant promise for transforming wastewater treatment into a more sustainable and effective process. By focusing on the critical areas of research and development outlined above, stakeholders can ensure that biochar is used efficiently and effectively, contributing to environmental sustainability and improved water quality.

To unlock the applicability of biochar in wastewater treatment on a larger scale, several areas warrant further investigation. (a) Pilot-scale validation: Transitioning from lab-scale studies to field trials will help assess long-term performance, regeneration, and scalability. (b) Feedstock optimization: Identifying regionally abundant, low-cost biomass sources can enhance sustainability and circular economy integration. (c) Hybrid systems: Combining biochar with solar-driven AOPs (advanced oxidation processes), IoT-enabled monitoring, or membrane technologies could yield synergistic effects in treatment efficiency. (d) Lifecycle and techno-economic analysis: Comprehensive assessments will guide policy, investment, and deployment strategies. (e) Policy integration: Establishing regulatory frameworks and incentives for biochar adoption will accelerate its uptake in environmental management.

## 6. Conclusions

The use of biochar in the removal of emerging inorganic and organic contaminants from water and wastewater has gained considerable traction in recent years. This review has provided a comprehensive overview of the mechanisms and techniques associated with biochar adsorption behavior, highlighting its effectiveness as a treatment option for various pollutants. Biochar stands out as a promising solution for addressing the challenges of water contamination. Continued research and innovation in this field will not only facilitate its practical applications but also contribute significantly to sustainable environmental practices and the remediation of polluted water systems. The versatile nature of biochar has rendered it a sustainable material for the remediation of both organic- and inorganic-type pollutants in wastewater. This review presented the multifaceted potential from the adsorption mechanisms to advanced modification strategies, positioning biochar as a key player in next-generation water treatment technologies in terms of cost and simplicity. Biochar is not merely an adsorbent, it proposes to be a bridge between agro-waste valorization, clean water access, and climate resilience. By combining interdisciplinary research and stakeholder collaboration, biochar-based technologies will play a transformative role in achieving SDG 6 (Clean Water and Sanitation), SDG 12 (Responsible Consumption and Production), and SDG 13 (Climate Action).

## Figures and Tables

**Table 1:** The removal efficiencies and adsorption capacities of biochar for various contaminants.

Contaminant	Biochar Source	Modification	Adsorption Capacity (mg/g)	References
**Lead (Pb)**	Sewage Sludge (SS), Rice Husk (RH)	Phosphate-solubilizing bacteria (PSB)	24.11–60.85	[78]
**Arsenic (As)**	Rice Husk	Bismuth-induced modification	16	[22]
**Mercury (Hg)**	Municipal Solid Waste	KOH treatment	101	[71]
**Tetracycline (TC)**	Wheat straw	Ball milling	99% removal	[57]
**Bisphenol-A (BPA)**	Straw	Alkali treatment	95–100% removal	[72]
**Indigo Carmine**	Olive Mill Waste	KOH treatment	599	[71]
**Methylene Blue**	Hickory Chips	Ball-milling, H_2_O_2_ treatment	High efficiency	[73,74,75,103]
**Nitrate, Phosphate, Arsenic**	Municipal Solid Waste	Alkaline and metal oxide modification	High removal efficiency	[66]
